# Supplementary material for: Developing and validating the Nepalese Abuse Assessment Screen (N-AAS) for identifying domestic violence among pregnant women in Nepal
Source: PLoS One. 2024 Jul 25;19(7):e0292563. doi: 10.1371/journal.pone.0292563 (PMC11271870; doi:10.1371/journal.pone.0292563)
Supplement: S1 Checklist — (DOCX) [file pone.0292563.s001.docx]

Inclusivity in global research

PLOS’ policy on inclusivity in global research aims to improve transparency in the reporting of research performed outside of researchers’ own country or community and ensures that PLOS publications reporting global research adhere to high standards for research ethics and authorship. Authors of relevant research articles may be asked to complete the questionnaire below, which outlines ethical, cultural, and scientific considerations specific to inclusivity in global research. This questionnaire may be requested when researchers have travelled to a different country to conduct research, if research uses samples collected in another country, research with Indigenous populations or their lands, or if research is on cultural artefacts. Researchers travelling to another country solely to use laboratory equipment will not normally be required to complete the questionnaire. However, the questionnaire can be requested at the journal’s discretion for any submission – if you have been requested to complete this questionnaire by the PLOS journal you submitted to, please do so.

Please complete the questionnaire below and include this as a Supporting Information file with your manuscript. Note that if your paper is accepted for publication, this checklist will be published with your article in the supporting information files. Please ensure that you reference the checklist in the main body of your manuscript. We suggest adding a subsection ‘Inclusivity in global research’ to your Methods section and adding the following sentence: “Additional information regarding the ethical, cultural, and scientific considerations specific to inclusivity in global research is included in the Supporting Information (SX Checklist)”

The questions have been designed to be applicable to a wide range of study types, and there are subsections for both human subjects research and non-human subjects research. If any of the questions are not relevant to your research please mark them as “N/A” as appropriate.

**Ethical considerations, permits and authorship**

*This section is applicable to all research types.*

Provide details as to who granted permissions and/or consent for the study to take place in the Methods section of your manuscript. This should include the names of **all** ethics boards, governmental organizations, community leaders or other bodies that provided approval for the study. If individuals provided approval refer to these people by their role or title but do not list their name(s).

Formal ethical approvals were obtained from The Nepal Health Research Council, Nepal (Ref: 2395) and The Regional Committee for Medical and Health Research Ethics of Central Norway (REK) (Ref: 178092). This is reported in the ‘Materials and methods’ section of the manuscript.

If there were any deviations from the study protocol after approval was obtained please provide details of these changes in the Methods section of your manuscript.

Not applicable (N/A)

Did this study involve local collaborators that are residents of the country where the research was conducted or members of the community studied? If you do not have any authors from said communities, please provide an explanation for this below.

Yes, this study involved local collaborators who are residents of Nepal, where the research was conducted. Five out of the 11 authors of this manuscript are residents of Nepal. These authors include the first two authors, who are PhD candidates actively working at the two collaborating hospitals where data for this study was collected, and, Poonam Rishal, a postdoctoral research working at both of the collaborating hospitals. Additionally, two of the authors, Sunil Kumar Joshi and Kunta Devi Pun, serve as local principal investigators, providing crucial insights and guidance throughout the research process.

Everyone listed as an author should meet PLOS’ criteria for authorship and all individuals who meet these criteria should be included in the author byline, rather than the acknowledgements. For further information please see the journal’s Authorship Policy.

**Human subjects research (e.g. health research, medical research, cross-cultural psychology)**

Did you obtain written informed consent from a representative of the local community or region before the research took place? How did you establish who speaks for the community? Details of written informed consent obtained from study participants should be reported separately in the Methods section of your manuscript.

Yes, prior to collecting data, formal ethical approvals were obtained the national ethics committee in the country where the research took place, the Nepal Health Research Council, Nepal (Ref: 2395). Regarding establishing who speaks for the community involved in the research, we took several steps to ensure representation and inclusivity. For example, Nepalese experts on women’s health and violence were included as topic experts in the validation study. The study collaboration includes several Nepalese health providers and researchers who educate health care providers in the country and are engaged in collaborations with community-based organizations and NGOs working for gender equality and health promotion. Our local authors/collaborators play integral roles in ensuring cultural sensitivity and representation throughout the research.

How did members of the local community provide input on the aims of the research investigation, its methodology, and its anticipated outcome(s)?

We organized a meeting with university and hospital Principals and Directors, Heads of the Gynae/Obs Departments and the Outpatient Wards to inform them about the research project, at both collaborating institutions. Following this, a project kick-off meeting was held, which included government staff from the Ministry of Women, Children, and Senior Citizens, police representatives, and members of the One Stop Crisis Management teams from various hospitals. We have provided opportunities for ongoing dialogue and feedback throughout the research process with doctors and antenatal care staff, allowing for their contributions and suggestions. They have assisted in various research tasks such as expediting patient registrations and providing patient assistance.

When engaging with the local community, how did you ensure that the informed consent documents and other materials could be understood by local stakeholders?

The informed consent document was written in straightforward, local language that was easily understood by community members. We conducted pretesting of the consent materials through Delphi rounds. Additionally, we provided verbal explanations of the consent documents along with written materials to ensure participant comprehension. Participants have noted that the written materials are clear, relevant, and do not raise questions about their intended meaning.

Will the findings of the research be made available in an understandable format to stakeholders in the community where the study was conducted (e.g. via a presentation, summary report, copies of publications, etc.)? Please provide details of how this will be achieved.

Yes, we will prepare reports and fact sheets of the key findings for local stakeholders (the two participating hospitals) and the government through presentations and distributing study publications. Health education programs on domestic violence will be conducted in various locations. The final PhD thesis dissertations resulting from this project will be shared at a dissemination conference, where we will invite and share key findings with government staff, hospital staff, and department personnel.

**Non-human subjects research using specimens/ animals collected as part of the study, or those housed in archival collections. Examples include archaeology, paleontology, botany and zoology.**

Did the permission you obtained from a local authority to perform the study include an agreement on access to outputs and benefit sharing? This may include procedures to enable fair distribution of the benefits and resources arising from the research performed. Please include any details of Prior Informed Consent and Benefit Sharing Agreements obtained. These may be required by field-specific regulations, for example the Convention on Biological Diversity (CBD) and the associated Nagoya Protocol.

N/A

If the material used in your study was imported, please A) provide the year it was imported and B) indicate whether permits were obtained to import/export the materials used, C) provide details of any permits obtained. If this information is not available, please indicate this.

N/A

If you used archival specimens, please state how the material used in your study was acquired by the institute it is held in and provide details of any permits obtained for the original excavations/ sample collection. If this information is not available, please indicate this.

N/A

How was the potential cultural significance of the materials collected in your study to local communities considered in your research design? Were Indigenous peoples and/or local researchers and institutions involved with archaeological excavations / collection of specimens? If so, please provide a description of their involvement.

N/A

If your manuscript includes photographs of human remains please indicate whether authors obtained permission from descendants or affiliated cultural communities to do so.

N/A
