# Supplementary figures and images for: Developing and validating the Nepalese Abuse Assessment Screen (N-AAS) for identifying domestic violence among pregnant women in Nepal
Source: PLoS One. 2024 Jul 25;19(7):e0292563. doi: 10.1371/journal.pone.0292563 (PMC11271870; doi:10.1371/journal.pone.0292563)

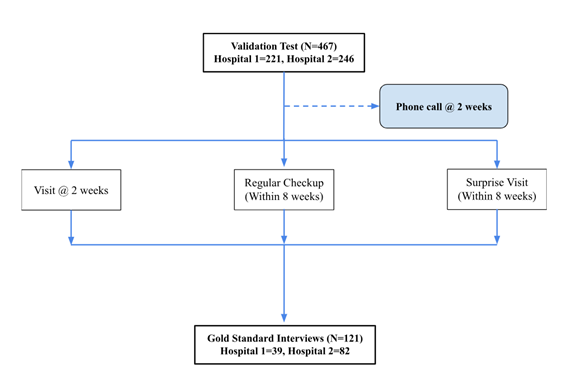

Supplement: S1 Fig — (TIF) [file pone.0292563.s002.tif]
