# Supplementary material for: Developing and validating the Nepalese Abuse Assessment Screen (N-AAS) for identifying domestic violence among pregnant women in Nepal
Source: PLoS One. 2024 Jul 25;19(7):e0292563. doi: 10.1371/journal.pone.0292563 (PMC11271870; doi:10.1371/journal.pone.0292563)
Supplement: S1 Table — (DOCX) [file pone.0292563.s003.docx]

**S1 Table. Test-retest reliability of N-AAS items (with Kappa coefficient).**

| **Question number** | **Total responses** | **Coefficient** | **Kappa value** |
| --- | --- | --- | --- |
| Q47.1_NAAS1 | 181 | 91.2% | 0.66 |
| Q48.1_NAAS2 | 181 | 97.8% | 0.70 |
| Q49.1_NAAS3 | 181 | 98.3% | 0.71 |
| Q50.1_NAAS4 | 181 | 98.3% | -0.00 |
| Q51.1_NAAS5 | 181 | 98.3% | 0.65 |
| Q52.1_NAAS6 | 181 | 98.3% | 0 |
| Q53.1_NAAS7 | 181 | 98.9% | 0.74 |
| Any violence | 181 | 91.2% | 0.69 |
