# Supplementary material for: Developing and validating the Nepalese Abuse Assessment Screen (N-AAS) for identifying domestic violence among pregnant women in Nepal
Source: PLoS One. 2024 Jul 25;19(7):e0292563. doi: 10.1371/journal.pone.0292563 (PMC11271870; doi:10.1371/journal.pone.0292563)
Supplement: S2 Table — (DOCX) [file pone.0292563.s004.docx]

**S2 Table. Test-retest reliability of comprehensive questionnaire (with Kappa coefficient).**

| **Question number** | **Total responses (181 minus missing)** | **Coefficient=(Same response/Total response)**^a^**100** | **Kappa** |
| --- | --- | --- | --- |
| Q1_FirstPregnancy | 181 | 99.4% | 0.98 |
| Q2_Deliveredbaby | 99 | 86.9% | 0.57 |
| Q3_LiveBoys | 93 | 91.4% | 0.83 |
| Q4_LiveGirls | 93 | 95.7% | 0.92 |
| Q5_DeathBaby | 99 | 90.9% | 0.69 |
| Q6_CS | 93 | 98.9% | 0.97 |
| Q7_FertilityTreatment | 181 | 95.0% | 0.44 |
| Q8_IN_FamilyPlanning | 181 | 81.8% | 0.49 |
| Q9_IN_BecomeMother | 181 | 94.5% | 0.64 |
| Q10_IN_Pregnant | 181 | 89.0% | 0.64 |
| Q11_IN_Baby | 181 | 80.1% | 0.54 |
| Q12_Preg_FamilyPressure | 181 | 97.2% | 0.27 |
| Q13_ANC | 181 | 91.7% | 0.27 |
| Q14_Tobacco | 181 | 100.0% | 1 |
| Q15_VigorousActivity | 181 | 91.7% | 0.30 |
| Q16_ModerateActivity | 181 | 84.5% | 0.32 |
| Q17_AGE | 181 | 87.3% | 0.81 |
| Q18_Ethnicity | 181 | 93.9% | 0.89 |
| Q19_Settlement | 181 | 95.6% | 0.89 |
| Q20_Education | 181 | 89.5% | 0.84 |
| Q21_IndependentIncome | 181 | 92.3% | 0.83 |
| Q22_PersonalAmount | 61 | 85.2% | 0.65 |
| Q23_PermissionIncome | 61 | 91.8% | 0.66 |
| Q24_FamilyType | 181 | 89.5% | 0.78 |
| Q25_Married | 181 | 98.3% | -0.00 |
| Q26_Widow | 0 | 0 |  |
| Q27a_Divored | 0 | 0 |  |
| Q27b_Separated | 0 | 0 |  |
| Q28_HavePartner | 0 | 0 |  |
| Q29_LiveHusband | 178 | 98.3% | 0.86 |
| Q29b_LivePartner | 0 | 0 |  |
| Q30a_SpanHusband | 165 | 86.7% | 0.80 |
| Q30.b_SpanPartner | 0 | 0 |  |
| Q31a_AgeHusband | 165 | 87.3% | 0.78 |
| Q31b_AgePartner | 0 | 0 |  |
| Q32a_AgeAtMarriageWithHusband | 165 | 87.9% | 0.80 |
| Q32b_AgeAtMarriageWithPartner | 0 | 0 |  |
| Q33a_HusbandAge | 165 | 81.8% | 0.71 |
| Q33b_PartnerAge | 0 | 0 |  |
| Q34a_OnlyWife | 165 | 98.2% | 0.39 |
| Q34b_OnlyPartner | 0 | 0 |  |
| Q35a_HusbandEducation | 165 | 84.8% | 0.77 |
| Q35b_PartnerEducation | 0 | 0 |  |
| Q36a_HusbandIncome | 165 | 90.3% | 0.64 |
| Q36b_PartnerIncome | 0 | 0 |  |
| Q37a_HusbandIncome | 130 | 90.0% | 0.54 |
| Q37b_PartnerIncome | 0 | 0 |  |
| Q38_FS1 | 181 | 87.3% | 0.28 |
| Q39_FS2 | 181 | 93.4% | 0.15 |
| Q40_FS3 | 181 | 94.5% | 0.23 |
| Q41_FS4 | 181 | 96.7% | 0.27 |
| Q42_FS5 | 181 | 97.2% | 0.25 |
| Q43_FS6 | 181 | 84.5% | 0.35 |
| Q44_FS7 | 181 | 73.5% | 0.31 |
| Q46_FS8 | 181 | 81.8% | 0.33 |
| Q47.1_NAAS1 | 181 | 91.2% | 0.66 |
| Q48.1_NAAS2 | 181 | 97.8% | 0.70 |
| Q49.1_NAAS3 | 181 | 98.3% | 0.71 |
| Q50.1_NAAS4 | 181 | 98.3% | -0.00 |
| Q51.1_NAAS5 | 181 | 98.3% | 0.65 |
| Q52.1_NAAS6 | 181 | 98.3% | 0 |
| Q53.1_NAAS7 | 181 | 98.9% | 0.74 |
| Under any Violence | 181 | 91.2% | 0.69 |
| Q54_Coping1 | 24 | 83.3% | 0.14 |
| Q55_Coping2 | 24 | 91.7% | 0.21 |
| Q56_Coping3 | 24 | 95.8% | 0.17 |
| Q57_Coping4 | 24 | 91.7% | 0.6 |
| Q58_Coping5 | 24 | 91.7% | 0.28 |
| Q59_Coping6 | 24 | 95.8% | 0.13 |
| Q60_Coping7 | 24 | 91.7% | 0.22 |
| Q61_Coping8 | 24 | 87.5% | 0.27 |
| Q62_Coping9 | 24 | 95.8% | 0.45 |
| Q63_Coping10 | 24 | 87.5% | 0.24 |
| Q64_Coping11 | 24 | 83.3% | 0.29 |
| Q65_Coping12 | 24 | 91.7% | 0.21 |
| Q66_Coping13 | 24 | 87.5% | 0.12 |
| Q67_Coping14 | 24 | 95.8% | 0.37 |
| Q68_Coping15 | 24 | 95.8% | 0.23 |
| Q69_Coping16 | 24 | 87.5% | 0.00 |
| Q70_Attitude1 | 181 | 94.5% | 0.46 |
| Q71_Attitude2 | 181 | 97.2% | 0.57 |
| Q72. I_Attitude3 | 181 | 87.3% | 0.54 |
| Q72.II_Attitude4 | 181 | 93.9% | 0.45 |
| Q73_Attitude5 | 181 | 93.9% | 0.36 |
| Q74_Attitude6 | 181 | 95.0% | 0.42 |
| Q75_Attitude7 | 181 | 94.5% | 0.47 |
| Q76_Attitude8 | 181 | 96.7% | 0.57 |
| Q77_Attitude9 | 181 | 96.7% | 0.48 |
| Q78_Attitude10 | 181 | 93.4% | 0.43 |
| Q79_Attitude11 | 181 | 93.9% | 0.51 |
| Q80_Attitude12 | 181 | 95.6% | 0.47 |
| Q81_Attitude13 | 181 | 95.6% | 0.54 |
| Q82_Attitude14 | 181 | 97.8% | 0.41 |
| Q83_Attitude15 | 181 | 91.7% | 0.36 |
| Q84_Attitude16 | 181 | 96.7% | 0.51 |
| Q85_GH1 | 181 | 97.2% | 0.40 |
| Q86_GH2 | 181 | 94.5% | 0.43 |
| Q87_GH3 | 181 | 84.5% | 0.46 |
| Q88_GH4 | 181 | 74.6% | 0.29 |
| Q89_GH5 | 181 | 80.7% | 0.26 |
| Q90_GH6 | 181 | 84.0% | 0.25 |
| Q91_GH7 | 181 | 85.6% | 0.35 |
| Q92_GH8 | 181 | 87.3% | 0.35 |
| Q93_GH9 | 181 | 91.2% | 0.37 |
| Q94_GH10 | 181 | 96.7% | 0.40 |
| Q95_HideMoney | 24 | 100.0% | 1 |
| Q96_HideKeys | 24 | 95.8% | 0.78 |
| Q97_Codes | 24 | 87.5% | 0.35 |
| Q98_CallPolice | 24 | 75.0% | -0.06 |
| Q99_RemovedWeapons | 24 | 79.2% | -0.06 |
| Q100_ToldSomeone | 24 | 45.8% | 0.06 |
| Q101_BirthCertificate | 24 | 91.7% | 0.62 |
| Q102._BanckAccount | 24 | 91.7% | 0.75 |
| Q103_Citizenship | 24 | 100.0% | 1 |
| Q104_DriverLicence | 24 | 75.0% | 0.53 |
| Q105_MarriageCertificate | 24 | 87.5% | 0.60 |
| Q106_Valuables | 24 | 79.2% | 0.54 |
| Q107_Phoneno | 24 | 87.5% | 0.65 |
| Q108_HiddenBag | 24 | 54.2% | 0.13 |
| Q109_Safehouse | 24 | 75.0% | 0.5 |
| Q110_Simcard | 24 | 91.7% | 0.76 |

^a^There were no reported instances or occurrences of physical abuse since pregnancy or food deprivation in the data.
